# Optimization of N-PERT Solar Cell under Atacama Desert Solar Spectrum

**DOI:** 10.3390/nano12203554

**Published:** 2022-10-11

**Authors:** Pablo Ferrada, Aitor Marzo, Miriam Ruiz Ferrández, Emilio Ruiz Reina, Benjamin Ivorra, Jonathan Correa-Puerta, Valeria del Campo

**Affiliations:** 1 Centro de Desarrollo Energético Antofagasta, Universidad de Antofagasta, Av. Angamos 601, Antofagasta 1270300, Chile; 2 Freelance Solar Energy Researcher, 04007 Almería, Spain; 3 MOMAT Research Group, Complutense University of Madrid, 28040 Madrid, Spain; 4 Supercomputing-Algorithms Research Group, University of Almería, 04120 Almería, Spain; 5 Department of Applied Physics II, Escuela de Ingenierías Industriales, Campus de Teatinos, University of Málaga, C/Doctor Ortiz Ramos, 29071 Málaga, Spain; 6 Interdisciplinary Mathematics Institute (IMI), MOMAT Research Group & Department of Applied Mathematics and Mathematical Analysis, Complutense University of Madrid, Plaza de las Ciencias 3, 28040 Madrid, Spain; 7 Departamento de Física, Universidad Técnica Federico Santa María, Av. España 1680, Valparaíso 11520, Chile; 8 Millenium Nucleus in NanoBioPhysics (NNBP), Av. España 1680, Valparaíso 11520, Chile

**Keywords:** photovoltaics, n-PERT, genetic algorithm, solar cells, drift-diffusion model, Atacama Desert, metallization

## Abstract

In the Atacama Desert, the spectral distribution of solar radiation differs from the global standard, showing very high levels of irradiation with a particularly high ultraviolet content. Additionally, the response of photovoltaic (PV) technologies is spectrally dependent, so it is necessary to consider local conditions and type of technology to optimize PV devices since solar cells are usually designed for maximum performance under standard testing conditions (STC). In this work, we determined geometrical and doping parameters to optimize the power of an n-type bifacial passivated emitter and rear totally diffused solar cell (n-PERT). Six parameters (the thicknesses of cell, emitter, and back surface field, as well as doping concentration of emitter, base, and back surface field) were used to optimize the cell under the Atacama Desert spectrum (AM 1.08) and under standard conditions (AM 1.5) through a genetic algorithm. To validate the model, the calculated performance of the n-PERT cell was compared with experimental measurements. Computed and experimental efficiencies showed a relative difference below 1% under STC conditions. Through the optimization process, we found that different geometry and doping concentrations are necessary for cells to be used in the Atacama Desert. Reducing the thickness of all layers and increasing doping can lead to a relative increment of 5.4% in the cell efficiency under AM 1.08. Finally, we show the potential effect of metallization and the viability of reducing the thicknesses of the emitter and the back surface field.

## 1. Introduction

Simulations of solar cells are very helpful to understand and predict the effect of fabrication parameters on the final performance [1,2]. In addition, they are very useful to assess the potential operation of photovoltaic (PV) devices under different environmental aspects, such as temperature or solar irradiance [3,4,5]. Furthermore, the cells’ geometrical and chemical properties, in terms of layer thicknesses, doping, and others, offer infinite possibilities, thus, optimization methods are required to improve devices.

Regardless of the simulation process, one of the most common ways to optimize parameters is through sweeping [6]. However, this method requires many simulations, given by the product of all the calculations performed for each free parameter. Conversely, genetic algorithms (GA) offer a more efficient path for optimization. These algorithms maximize a given objective, finding the optimal combination of variables without sweeping all possibilities, which significantly reduces computational requirements and time. This is possible through the emulation of natural selection, implementing selection, crossover, and mutation for consecutive generations until optimum is achieved [7].

Genetic algorithms were successfully used to find optimal design parameters for PV devices [6,8,9]. Vincent et al. [6] showed that using a GA is more accurate and significantly faster than the sweeping method for a multi-layer optimization of organic solar cells. They were able to reduce the number of simulations by 60% when starting with the correct parameters. If the initial parameters were not good enough, the GA needed a similar number of simulations, compared to sweeping, but never more than sweeping. Razzaq et al. [8] used a genetic algorithm combined with the rigorous coupled-wave analysis method to improve perovskite/c-Si tandem cells. They optimized five interdependent parameters (related to optical elements) and were able to maximize absorption in the perovskite and in the c-Si absorber layer. Attari et al. [9] optimized the design of GaAs solar cells through GA. They tested thicknesses and doping of the five layers of the cell at the same time, resulting in the ideal junction configuration for the highest efficiency.

In crystalline silicon (c-Si) solar cells, bifaciality has gained great interest, offering the possibility to increase production per unit area by 20% to 30% [10,11]. In particular, the n-type bifacial passivated emitter and rear totally diffused solar cell (n-PERT) have achieved a bifaciality of 95%, showing efficiencies of 22% under standard conditions [12].

Spectral irradiance is a key parameter in the performance of solar devices, especially those that have a spectrally dependent response. Since spectral distribution of solar irradiance can vary markedly over time and from place to place, there exists an important need to analyze the potential performance of PV technologies under local solar spectral irradiance [13,14,15,16,17].

In the present work, we determined the doping and geometrical parameters of an n-PERT solar cell to optimize its power output. The cell was modeled based on the drift-diffusion and continuity equations. Through a genetic algorithm, we were able to optimize the cell thickness, base doping, emitter depth and doping concentration, and thickness and doping of the back surface field, considering a representative Atacama Desert solar spectrum (AM 1.08) and separately under the standard reference spectrum (AM1.5G). We compared the obtained parameters with the actual parameters of the solar cells and showed how they change depending on the spectrum. We also discussed the effect that metallization could have on the obtained thicknesses and cell performance.

## 2. Materials and Methods

The approach to accomplish the goals of this work consists of two steps: validation of the model and cell optimization. The validation consists of comparing experimental measurements of an n-PERT cell with the model’s numerical results under the reference spectra in both cases. The optimization consists of finding optimal values in the selected parameters for maximum performance when illuminated with the mean Atacama Desert solar spectrum (AM1.08) and when illuminated with the AM1.5G standard spectrum.

Originally, the standard reference spectrum was created in 1992 from spatio-temporal averages of one-year databases of atmospheric parameters for the United States of America (U.S.A.). From these averages and using the SMARTS [18,19] radiative transfer model, standard spectra were obtained for the direct normal and global tilted (at 37°) spectral irradiances under 1.5 airmass. The 37° slope of the inclined surface facing the sun was chosen to represent the average latitude of the 48 contiguous United States. In the latest version of the 2020 standard, they make the SMARTS model available (as a complement (ADJG173CD 3) to the standard).

**Figure 1:**
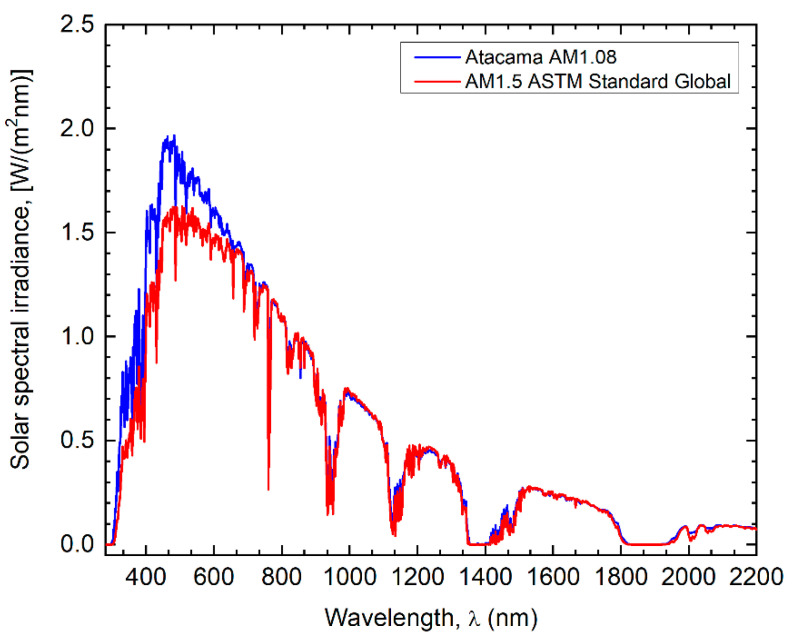
Atacama Desert AM1.08 (blue line) and reference AM1.5G (red line) solar spectra.

In 2018, the mean solar spectra for the Atacama Desert were calculated from the spatio-temporal averages of the long-term databases of local atmospheric parameters [13]. The long-term databases contained satellite and model retrievals data, from MODIS [20,21,22], AIRS [23], provided by Era Interim [24,25] and Giovanni [26] for the extent of the Atacama Desert in Chilean territory. In order to replicate the methodology used for the development of the standard reference spectrum, the SMARTS model [18,19] and the spatio-temporal averages of the local atmospheric parameters were used. Furthermore, the 18° slope of the inclined surface facing the sun was chosen to represent the average latitude of the Atacama Desert between 13°S and 30°S. Finally, for the mean airmass value calculation, the median mean airmass at noon was calculated for the same latitudes resulting in a mean airmass of 1.08. All of this allowed us to obtain the mean solar spectra for the Atacama Desert with a methodology similar to that of the standard reference spectrum. Further details are published in Marzo et al. [13]. See Figure 1.

### 2.1. Model Validation at STC

**Figure 2:**
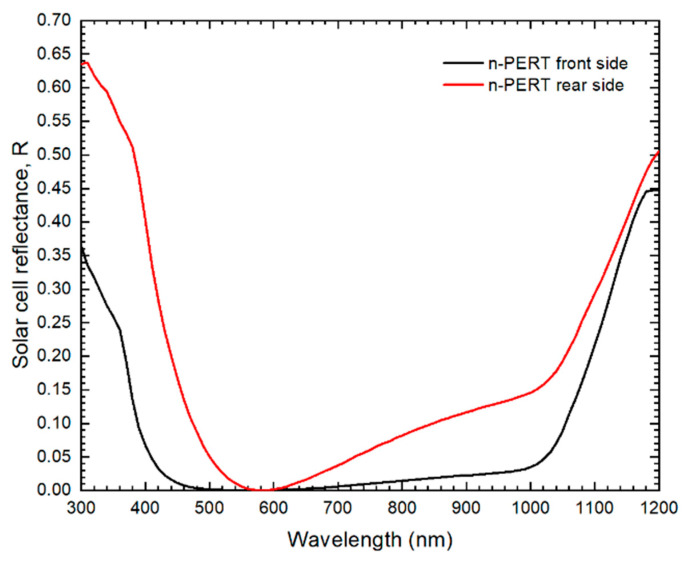
Spectral reflectance of the n-PERT solar cell [3].

For validation, we used the experimental characterization of n-PERT solar cells, published in Ferrada et al. [27]. Table 1 shows the geometrical, doping and Shockley-Read-Hall (SRH) parameters of the cell, while the output current–voltage characteristics are shown in Table 2 (in the Results section). Values for the SRH carrier lifetimes were obtained from Edler [18] and Fell et al. [19] [1,28], while the metal fraction, $f_{met}$*,* corresponds to the ratio of the cell’s front surface covered by metallization. In addition, the spectral reflectance (*R_λ_*) of the metallized solar cell, shown in Figure 2, was obtained using a Perkin Elmer 950 Spectrophotometer [3]. See the Appendix A for the full parameter list.

A 1D model (detailed in the Theory section) was developed in the semiconductor module of COMSOL Multiphysics v6 and tested in laboratory under STC considering AM1.5G reference spectra. Once the current–voltage characteristics (IV) were calculated, electrical output parameters, such as the short circuit current density ($J_{sc}$), open circuit voltage ($V_{oc}$), fill factor (FF), power ($P_{mpp}$, where *mpp* stands for maximum power point), and efficiency (η), could be compared to the measured IV parameters. Note that in this work, current density is used instead of current. A summary of the measured and calculated JV curves under STC is shown in Table 2 of the results section.

**Figure 3:**
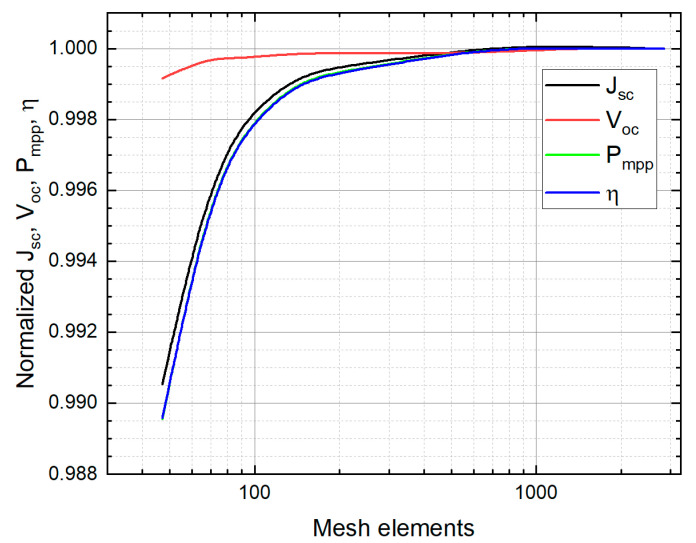
Mesh independence study of the main output JV parameters as a function of the mesh elements for a 1D model.

A mesh independence study was conducted to identify the minimum number of elements, which led to satisfactory results. That is, an output parameter obtained with a certain number of mesh elements did not vary significantly for more mesh elements. The analysis was applied to $J_{sc}$, $V_{oc}$, $P_{mpp}$, and η. The mesh study is shown in Figure 3, where the electrical parameters are normalized to those using the largest number of mesh elements. The analysis indicates that above 600 mesh elements, the output parameters match almost perfectly with the parameters computed using the extremely fine mesh. A fine mesh (which means 210 elements) can be used since it produces a variation only at the third significant figure.

To compare the model with experimental results, two main factors were considered:

(1) The model assumes flat surfaces; thus, a correction factor must be introduced to match the short current density. However, the increase in surface area is accompanied by an increment in the saturation current density $J_{0}$ due to recombination. Fell et al. [1] considered both issues and defined correction factors ($f_{corr}$) for different solar cells. In this work, the calculated short current density under STC conditions, $J_{sc,cal}$, was multiplied by $f_{corr}=1.17$ for comparison with the measured short current density, $J_{sc,meas}$.
(2) The model does not consider metal induced recombination. Therefore, the open circuit voltage $V_{oc}$ may be higher than the experimental result. These differences were quantified and fully described by Edler et al. [29]. They quantified the reduction in the open circuit voltage $\Delta V_{oc}$ of n-PERT solar cells by varying the metal fraction $f_{met}$ and quantifying the dark saturation current density at the metal/semiconductor interfaces. For the metal fraction of n-PERT solar cells in this work (0.099 and 0.052 for the front and rear side, respectively), the estimation indicates that the $V_{oc}$ can decrease 45 mV due to the front side metallization and 35 mV due to the rear side metallization. It is pointed out that the effect of series and shunt resistances, $R_{ser}$ and $R_{shunt}$, is observable in the shape of the IV curve, and thus, on the FF and $P_{mpp}$ [30]. Based on the referenced experimental work, metal induced recombination can be applied after the model is solved, by subtracting $\Delta V_{oc}$ for the corresponding metal fraction. In addition, ohmic losses will affect $P_{mpp}$ and FF. For power, $P_{mpp}=P_{mpp,0}-R_{ser}I_{mpp}{}^{2}$, where $P_{mpp,0}$ is the power without resistance effects. For the fill factor, $FF=FF_{0}\left(1-r_{ser}\right)$, where $FF_{0}$ is the fill factor without resistive effects, $r_{ser}$ is the normalized series resistance to the characteristic resistance ($r_{ser}=R_{ser}/R_{CH}$ and $R_{CH}=V_{oc}/I_{sc}$) [31].

### 2.2. Determination of the Optimal Solar Cell Parameters

**Figure 4:**
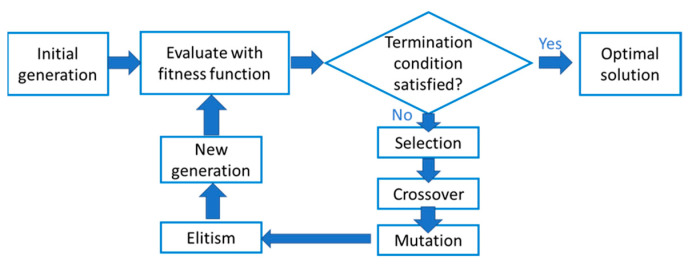
General procedure of the considered genetic algorithm.

Six optimization parameters were defined to maximize the output power of the solar cell. These parameters are the cell, emitter, and back surface field thicknesses ($d_{cell},\;\;d_{E},\;$and $d_{BSF}$, respectively) as well as emitter, base, and BSF doping concentration ($N_{E}$, $N_{B}$, and $N_{BSF}$, respectively). To find the optimal parameters, we used a particular genetic algorithm (GA) with the following stages (described in detail by Katoch et al. [7]); see Figure 4.

Stage 1

First, we randomly produced a first generation of individuals in the search space Ω. That means, given a fixed population size $N_{p}\in \mathbb{N}$, we randomly generated $N_{p}$ points: $X^{0}=\left\{x_{1}^{0},\;\ldots ,x_{N_{p}}^{0},\;\mathrm{in}\;\mathrm{such}\;\mathrm{way}\;\mathrm{that}\;x_{1}^{0}\in \Omega ,\mathrm{with}\;i=1\ldots N_{p}\right\}$, where $X^{0}$ is called the initial generation (or generation 0).

Stage 2

During a fixed number of iterations $N_{g}\in \mathbb{N}$, called generations, several sub processes were repeated. For each generation $n=0\ldots N_{g}$, considering $X^{n}$, we applied the following processes.

- Selection: We calculated the value of the cost function g of all points $x_{i}^{n}$, and it was denoted as $g_{i}^{n}=g\left(x_{i}^{n}\right)$, with $i=1,\ldots N_{p}$. For each $x_{i}^{n}$, a probability $p_{i}^{n}$ to be selected, was assigned. The probability can be written in terms of the cost function, $p_{i}^{n}=p\left(g_{k}^{n}\right)$. Once the probability was computed, $2N_{p}$ elements called parents were randomly chosen, and they were denoted as $y_{i}^{n}$, with $i=1,\ldots 2N_{p}$. This procedure assumed that the region of the points with the lowest value of g is explored with a larger frequency.
- Crossover: We created $N_{p}$ elements called children and denoted as $e_{i}^{n}$, with $i=1,\ldots N_{p}$, from the values of the parents $y_{i}^{n}$ by considering a random point included in the segment defined by two parents. This step was intended to explore a zone included between two parents’ points and determine if there was a better element.
- Mutation: We modified randomly some components of the $e_{i}^{n}$ elements. The goal was to explore some areas of the search space randomly. In addition, this step allowed for escape from possible local minima, which may attract too many elements of the population.
- Elitism: We aimed to ensure that the convergence of the GA was always decreasing, that is, the value of g of the best element from each generation was decreasing from one generation to another. Thus, we directly copied the best element from the previous generation $X^{n}$ and it was denoted as ${\bar{x}}^{n}$.

After implementing these steps, the next generation $X^{n+1}=\left\{e_{1}^{n},\ldots ,e_{N_{p}}^{n}\right\}$ was obtained. Once the GA was finished, it led to the point ${\bar{x}}^{n}$, where n was the last calculated generation. This solution is an approximation for the optimization problem. In general, this algorithm can rapidly find a zone near the global optimum. However, it can lack of precision [32].

Once the algorithm was defined, the parameters: $N_{p}$, $N_{g}$, $p_{m}$, were chosen. Additionally, the stopping tests were defined. If, during $N_{s}$ iterations, the value of ${\bar{x}}^{n}$ remained unchanged, the GA was stopped. Once the solution was obtained, a convergence curve was constructed. Finally, the performance of the solar cell was computed using the optimized parameters for each solar spectrum. In this case the model was executed under the Atacama AM1.08 and the standard reference spectrum AM1.5G.

## 3. Theory

The JV curve of the c-Si cells can be calculated through the following model based on: (i) net charge relation, (ii) holes and electrons transport, and (iii) continuity equations.

i. Net charge density relation

The net charge density relation is $\nabla \cdot \left(-\varepsilon_{r}\nabla V\right)=\rho$, where $\varepsilon_{r}$ is the relative permittivity of the semiconductor, V is the electric potential, and ρ is the charge density given by $\rho =q\left(p-n+N_{d}^{+}-N_{a}^{-}\right)$, in which q is the elementary charge, n and p are the electron and hole concentration, and $N_{d}^{+}$ and $N_{a}^{-}$ the ionized impurity concentration. See Equation (1).

(1) $$\nabla \cdot \left(-\varepsilon_{r}\nabla V\right)=\rho =q\left(p-n+N_{d}^{+}-N_{a}^{-}\right)$$

ii. Transport equations

The transport of electrons and holes in terms of the current densities $J_{n}$ and $J_{p}$, are shown in Equations (2) and (3), respectively. The quantities $\mu_{n}$ and $\mu_{p}$ are the electron and hole mobilities and $E_{C}$ and $E_{V}$ are the conduction and valence band edges. In addition, $k_{B}$ is the Boltzmann’s constant, T is the temperature, $N_{C}$ and $N_{V}$ are the effective density of states in the conduction and valence bands and G is the ratio of the Fermi integrals, $G\left(n/N_{C}\right)={ℱ}_{1/2}\left(\zeta \right)/{ℱ}_{-1/2}\left(\zeta \right)$ with $\zeta ={ℱ}^{-1}{}_{1/2}\left(n/N_{C}\right)$. In the last terms, $D_{n,th}$ and $D_{p,th}$ correspond to the thermal diffusivities.

(2) $$J_{n}=qn\mu_{n}\nabla E_{C}+\mu_{n}k_{B}TG\left(\frac{n}{N_{C}}\right)\nabla n+qnD_{n,th}\nabla \ln\left(T\right)$$

(3) $$J_{p}=qp\mu_{p}\nabla E_{V}-\mu_{p}k_{B}TG\left(\frac{p}{N_{V}}\right)\nabla p-qpD_{p,th}\nabla \ln\left(T\right)$$

The first and second terms in Equations (3) and (4) stand for the drift and diffusion model for electrons and holes; whereas the third term corresponds to corrections related to Fermi–Dirac statistics [33]. Carrier mobilities, affected by scattering of charge carriers due to impurities and phonons, are computed through the empirical Arora mobility model [34] (Equation (4)). The Arora model includes the dependency on the impurities but also on the cell temperature through $\mu_{min}$, $\mu_{0}$, and m, which are *T*-dependent (details in [35]). See the Appendix A for the extended form of the Arora model equations.

(4) $$\mu =\mu_{min}+\frac{\mu_{0}}{1+{\left(N/N_{ref}\right)}^{m}}$$

iii. Continuity equations

The continuity equations for electron and holes are Equations (5) and (6), where $U_{n}$ and $U_{p}$ are the net electron and hole recombination rates, given by $U_{n}=\sum R_{n,i}-\sum G_{n,i}$ and $U_{p}=\sum R_{p,i}-\sum G_{p,i}$, where R and G denotes recombination and generation, respectively

(5) $$\frac{\partial n}{\partial t}=\frac{1}{q}\left(\nabla \cdot J_{n}\right)-U_{n}$$

(6) $$\frac{\partial p}{\partial t}=-\frac{1}{q}\left(\nabla \cdot J_{p}\right)-U_{p}$$

The generation rate G is obtained through the Lambert–Beer’s law: $\phi \left(\lambda ,z\right)=\phi_{0}e^{-\alpha \left(\lambda \right)\;z}$, where ϕ(λ,z) is the number of photons of wavelength λ per unit area and time at a given point in the cell. The term $\phi_{0}$ corresponds to the incident photon flux, α is the absorption coefficient (in cm^−1^), and z the spatial coordinate. Assuming that each photon leads to an electron–hole pair, dismissing reflection, the generation rate $G_{0}$ (in cm^−3^s^−1^) is computed as the derivative of ϕ(λ,z) with respect to z and integrating over λ (Equation (7)). A similar process was used by Chowdhury et al. [36].

(7) $$G_{0}\left(z\right)=\int_{0}^{\infty }\alpha \left(\lambda \right)\phi \left(\lambda \right)e^{-\alpha \left(\lambda \right)\;z}d\lambda$$

The absorption coefficient is given by α(λ)=4πκ(λ)/λ, where κ(λ) is the extinction coefficient. The photon flux is linked to the solar spectral irradiance, F(λ) via ϕ(λ)=λF(λ)/(hc), where h is Planck’s constant and c the speed of light in vacuum. The next step is to consider the metallized fraction of the cells, $f_{met}$, and the spectral reflection of the non-metallized region of the solar cell, R(λ). Then, the generation rate becomes Equation (8):

(8) $$G\left(z\right)=\frac{4\pi }{hc}\left(1-f_{met}\right)\int_{\lambda_{1}}^{\lambda_{2}}\kappa \left(\lambda \right)\;F\left(\lambda \right)\;e^{-\frac{4\pi \;\kappa \left(\lambda \right)\;z}{\lambda }}\left[1-R\left(\lambda \right)\right]d\lambda$$

Losses due to Auger ($R_{Auger})$, trap-assisted (Shockley–Read–Hall, $R_{SRH}$) and band-to-band ($R_{D}$) recombination are included through Equations (9)–(11). In these equations, $C_{n}$,$C_{p}$, and C are material constants; $\gamma_{n}$ and $\gamma_{p}$ are degeneracy factors obtained in terms of Fermi integrals; $n_{i,eff}$ is the intrinsic carrier concentration; $\tau_{p}$ and $\tau_{n}$ are hole and electron lifetimes, respectively; and $p_{1}$ and $n_{1}$ are hole and electron expressions, depending on the trap energy level $E_{t}.$ The intrinsic carrier concentration is obtained via $n_{i,eff}{}^{2}=N_{C}N_{V}\;e^{-E_{g}/\left(k_{B}T\right)}$, where $E_{g}$ is the semiconductor’s bandgap.

(9) $$R_{Auger}=\left(C_{n}+C_{p}\right)\left(np-\gamma_{n}\gamma_{p}n_{i,eff}^{2}\right)$$

(10) $$R_{SRH}=\frac{np-\gamma_{n}\gamma_{p}n_{i,eff}^{2}}{\tau_{p}\left(n+n_{1}\right)+\tau_{n}\left(p+p_{1}\right)}$$

(11) $$R_{D}=C\left(np-\gamma_{n}\gamma_{p}n_{i,eff}^{2}\right)$$

The $n_{1}$ and $p_{1}$ are defined as Equations (12) and (13) show, where $\Delta E_{g}$ is the band gap narrowing energy, $V_{th}$ is the thermal voltage, and $E_{t}$ is the trap energy level.

(12) $$n_{1}=\gamma_{n}\sqrt{N_{C}N_{V}}exp\left(-\frac{E_{g}-\Delta E_{g}}{2V_{th}}\right)\;exp\left(-\frac{\Delta E_{t}}{V_{th}}\right)$$

(13) $$p_{1}=\gamma_{n}\gamma_{p}\sqrt{N_{C}N_{V}}exp\left(-\frac{E_{g}-\Delta E_{g}}{2V_{th}}\right)\;exp\left(-\frac{\Delta E_{t}}{V_{th}}\right)$$

## 4. Results

### 4.1. Measurements and Simulation of the Solar Cell at Standard Conditions 

The validation step is performed through a direct comparison between the measured and computed JV parameters. The obtained values from both methods are presented in Table 2. Measurements were performed for the front and rear side of a set of six solar cells leading to the standard deviations indicated in the Table 2. The area of the measured solar cells was 244.3 cm^2^. Using the value of the series resistance $R_{ser}=0.45\;\Omega {\mathrm{cm}}^{2}$ from the experimental measurements, the following results were obtained.

According to the values in the table, relative differences ΔX for a parameter X were determined as $\Delta X=(X_{calc}-X_{meas})/X_{meas}$, where $X_{calc}$ and $X_{meas}$ stand for calculated and measured, respectively. The $J_{sc}$ and $V_{oc}$ show relative differences between calculated and measured values below 1% for the front side and below 2% for the rear side. The power $P_{mpp}$ is the parameter experimenting the largest discrepancy between measured and calculated values, being 8% for the front and 7% for the rear side. However, the FF and efficiency exhibit relative differences below 1% for both sides. In summary, the error of the simulated JV parameters is below 2%, except for maximum power, which reached values up to 8% when illuminated with the reference solar spectrum AM1.5G. Since the maximum power point is calculated considering the series resistance through $P_{mpp}=P_{mpp,0}-R_{ser}I_{mpp}{}^{2}$, the corresponding error $\delta P_{mpp}$ includes the value of $R_{\mathrm{ser}}$ and the propagation of the short current density error, $\delta J_{sc}$. With $R_{\mathrm{ser}}=0.45{\;\Omega \mathrm{cm}}^{2}$, the propagation error can reach 16%, which explains the discrepancy of 8% with the measurement.

### 4.2. Optimal Solar Cell Parameters

As stated in the methodology, the optimization aimed to obtain maximum output power $P_{mpp}$ for front side illumination at the AM1.5G standard and the AM1.08 spectra through the selected control parameters. The initial values and range for these parameters are summarized in Table 3. The parameters of the GA are found in Table 4.

**Figure 5:**
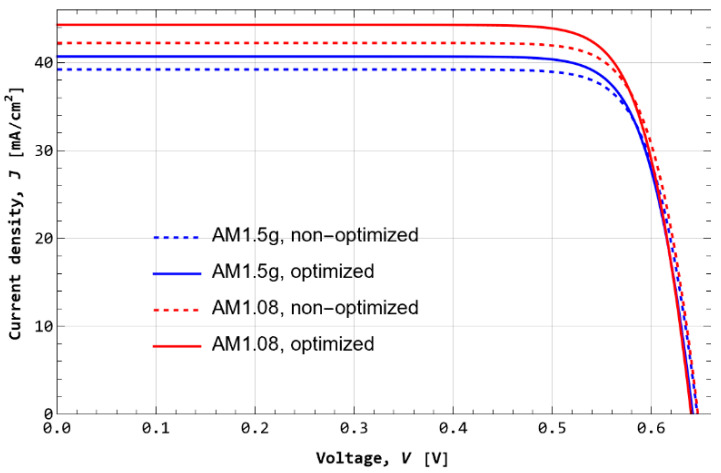
Current–voltage curve of the optimized and non-optimized n-PERT solar cell at the AM1.5G and AM1.08 spectra.

Figure 5 shows the simulation of the optimized (full line) and non-optimized (dotted curve) n-PERT solar cell at the AM1.5G (blue) and AM1.08 (red) solar spectra. The calculation shows that for both spectra, the short circuit current density of the optimized solar cell is increased with respect to that of the non-optimized cell, while keeping the open circuit voltage nearly at the same value. Thus, the power output is also increased. The implications of this result suggest that the global optimization, performed through a genetic algorithm, was successful in finding solar cell parameters that increase the power output though the current density and reducing recombination.

Based on the JV curves, Table 5 shows the JV parameters of the optimized and the non-optimized cases for both spectra; and the relative increment after optimization The relative percentual increment in each electrical parameter *X* obtained from the optimization is computed as $100\left(X_{opt}-X_{non-opt}\right)/X_{non-opt}$. The terms $X_{opt}$ and $X_{non-opt}$ refer to optimal and non-optimal values. Under both spectra, optimized cells show increments in all parameters except for the open circuit voltage, as previously observed in the IV curves. The largest improvements due to optimized parameters are observed under the Atacama spectrum, reaching an increment of 5.7% in the maximum power and of 5.4% in efficiency. This is expected since solar cells are optimized for STC.

Table 6 shows the obtained geometrical and doping parameters for the optimized solar cell under both spectra in comparison with the experimental values of the n-PERT device. The optimal thickness found for the emitter is in both cases thinner than the initial case (approximately 1/3). This fact may increase the probability of recombination caused by the minority carrier diffusion towards the unpassivated interface, leading to a lower open circuit voltage [37]. On the other hand, making a thinner emitter means that at the same temperature (to produce *d*_E_ = 650 nm or *d*_E_ = 200 nm), a shorter time is required [38]. In the case of the thickness of the BSF, the same reasoning as above follows. Regarding the thickness of the silicon wafer (cell thickness), optimal values are smaller than the initial one. The use of thinner wafers implies a more efficient use of material (cost reduction) and the possibility of better heat management under illumination [39]. Additionally, the voltage benefits from a lower thickness [40].

Table 6 indicates that the emitter doping concentration is slightly lower at AM1.08 compared to that at the standard spectrum. This result can be interpreted as a requirement to reduce recombination caused by the high-power density spectrum in Atacama. A higher intensity means a larger number of photons impinging the solar cell, significantly increasing the excess minority carrier density and, thus, the Auger recombination [41] at the front side. Therefore, reducing the doping concentration is needed to keep recombination low enough.

Table 6 also shows that the thickness of the BSF is thinner and heavier doped at AM1.08 compared to the value at STC. The BSF was linked to recombination reduction at the rear side through a junction of same polarity (*n*-*n*^+^) in this case [42] (where the “+” denotes higher doping concentration). This result for the BSF is inverse to that at the emitter because photons are mostly absorbed in the emitter and base. Thus, limiting doping concentration at the rear side is not necessarily due to a lower excess of minority carrier density compared to the front side, allowing a thinner and heavier doped BSF. Studies regarding the optimal thickness for a phosphorus doped layer, such as the BSF of the n-PERT solar cell, indicate that for low doping concentration, the thickness is higher compared to that at higher doping concentration, where thickness needs to be limited to avoid recombination [43]. Stem and Sid [43] found that the optimal thickness for the P-doped layer is below 1 µm for doping concentrations from 5 × 10^19^ cm^−3^ to 1 × 10^20^ cm^−3^, reaching highest efficiency in the range of 0.2 to 0.4 µm. 

## 5. Discussion

### 5.1. Solar Spectrum

Our findings show that the solar spectrum has an important effect on the cell performance. The solar cell shows a different response under the Atacama Desert spectrum, due to the larger ultraviolet content (blueshift) and its higher intensity compared to AM 1.5G. Thus, the optimal set of parameters under a local spectrum, i.e., the AM 1.08, are different compared to AM 1.5G illumination. Cells fabricated for the Atacama Desert could use thinner Si wafers, with lower thicknesses for the base, the emitter, and BSF. At the same time, emitter doping has to increase by ~4 times and BSF doping by ~8 times. Through this optimization, the specially designed cell could further increase the performance of PV devices under the Atacama spectrum reaching efficiencies of 22.7% instead of the 21.6% computed for the non-optimized cell and of the 20% measured at STC.

The increment in the short current density under the Atacama Desert spectrum agrees with previous works [3,13,44,45]. In Marzo et al. [13], two c-Si solar cells with different sheet resistance, and showing different external quantum efficiencies, were evaluated under both spectra by means of the photogenerated current density ($J_{ph}$). Equation 14 corresponds to the model used in that work, where q is the elementary carrier charge, h is the Planck’s constant, c is the speed of light in vacuum, EQE is the external quantum efficiency, and F is the solar spectral irradiance.

(14) $$J_{ph}=\frac{q}{hc}\int_{\lambda_{1}}^{\lambda_{2}}EQE\left(\lambda \right)F\left(\lambda \right)\lambda d\lambda$$

In that case, under AM 1.08, the increment in $J_{ph},$ due only to the UV range, was in the order of 50%. While, in the visible (VIS) and infrared (IR) spectral ranges, it also led to an increase of 10% and 1.4%, in the photocurrent density. The main difference between both solar cells was a larger blue response in the one with 100 Ohm/sq vs. the other with 50 Ohm/sq sheet resistance. A similar calculation was performed for five c-Si technologies under the standard and Atacama spectra [3]. Additionally, the effects of different encapsulant materials were evaluated, and in all cases, $J_{ph}$ was larger in the Atacama Desert compared to the standard spectrum. A different insight into the discussion regarding the impact of the Atacama AM1.08 solar spectrum was provided by Diaz et al. [44]. Their approach enabled the estimation of the maximum photogenerated current density in terms of internal parameters of the cell and its optical performance. For this purpose, an effective optical pathlength enhancement factor, Z(λ), was introduced. The methodology was applied for crystalline silicon devices and for dye-sensitized solar cells under the standard AM 1.5G (with cells tilted 37°) and the Atacama AM1.08 (with cells tilted 18°). In the case of Si technologies, there is an important photocurrent increase under AM1.08, while the difference is small for dye-sensitized solar cells. An experimental work [45] with indoor (STC) and outdoor (Atacama) measurements performed on c-Si cells showed different results. In the experiment, under outdoor conditions in the Atacama Desert, the short circuit current was 2.8% lower than under indoor (standard) testing. The authors explain the result is due to the greater resistance effects from cables and contacts in the outdoor experiment, which was observed in a 10% larger fill factor.

**Figure 6:**
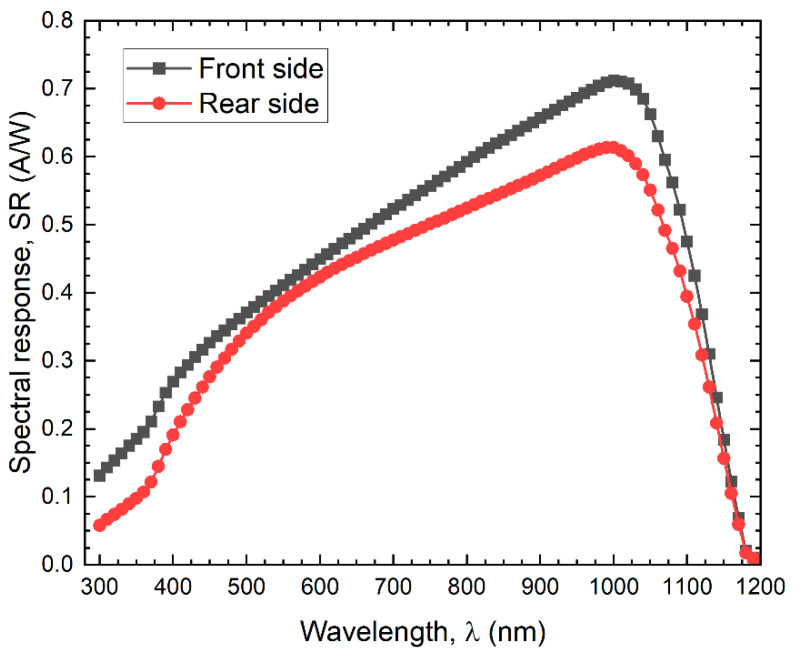
Spectral response of an n-PERT solar cell, measured for the front and rear sides under 1 sun.

Figure 6 shows the spectral response in the studied cell. The average SR values for the front side are 0.19, 0.44, and 0.53 A/W in the UV, VIS, and in the IR spectral range. Similarly, for the rear side, average values are 0.11 A/W, 0.40 A/W, and 0.46 A/W, respectively. Through this measurement, we can calculate the photogenerated current density, $J_{ph}$, and compare it with the measured $J_{sc}$ and with the computed $J_{sc}$ obtained with COMSOL. The equation to obtain $J_{ph}$ when SR is known is expressed in Equation (15).

(15) $$J_{ph}=\int_{\lambda_{1}}^{\lambda_{2}}\mathrm{SR}\left(\lambda \right)F\left(\lambda \right)d\lambda$$

Table 7 shows the measured and calculated current density for front and rear sides of the cell under both spectra. For the front side, through the COMSOL calculation, the same value of the measurement is obtained, while there is a relative difference of 0.8% for the calculated $J_{ph}$. In the case of rear-side illumination, the relative differences between the measurements are 1.2% for Jsc via COMSOL and 0.9% for the calculated $J_{ph}$.

### 5.2. Metallization

With the aim to analyze the feasibility of the optimal parameters found for the solar cell, the focus of this section is to look deeper into the metallization step, which was not implemented in the optimization but explored experimentally. For this purpose, characterization and modeled results regarding the metallization step are considered.

**Figure 7:**
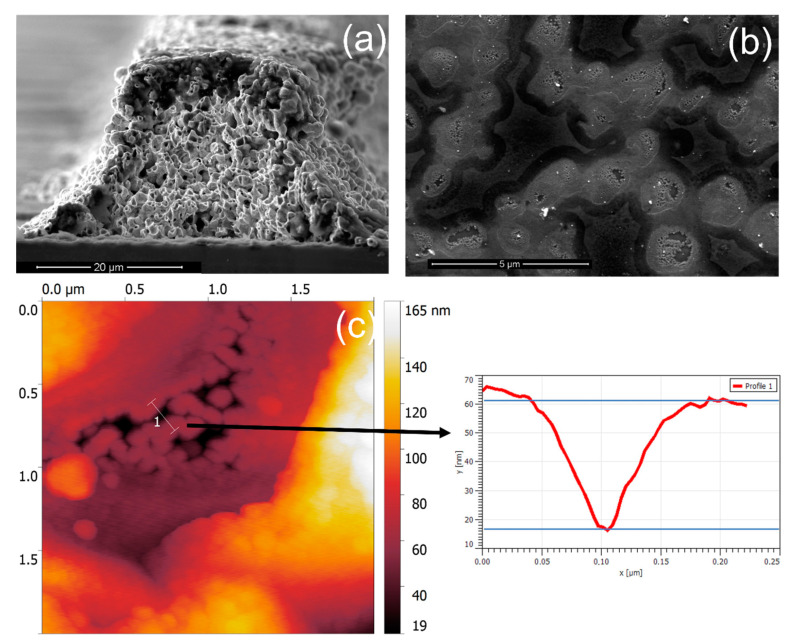
(**a**) SEM image of a silver finger of the n-PERT solar cell with a width around 50 µm and height of 10 µm after a single print. This structure was removed for the interface analysis. (**b**) Image of the SiNx etched area due to metallization; the light gray areas correspond to the etched areas (for further details, see [27]). (**c**) AFM image and depth profile of Ag imprints left behind after the chemical treatment.

To experimentally investigate the effect of metallization and doping concentration, we chose the rear side of n-PERT solar cells because the flat surface facilitates characterization (see Ag finger in Figure 7a). During metallization, silver pastes etch the SiNx layer, and Ag crystallites penetrate the BSF [27]. Both processes influence the cell performance. The etched area due to the action of the glass frit of the metallization paste was determined by field emission scanning electron microscopy (FE-SEM), followed by image analysis (Figure 7b). The surface was prepared using a HNO_3_ + HF + HNO_3_ treatment to remove the fingers’ glass and silver and allow the etched SiNx area to be visible. It was found that the etched area can vary between 51% and 67% depending on paste composition. The more aggressive paste led to poorer solar cell performance, expressed in the efficiency. In both cases, this etching process meant that the passivation underneath the metal contact was removed, leading to an enhanced recombination at the metal–Si interface [29]. Penetration of Ag crystallites is the main contributor to the current transport between Ag and c-Si [46]. Based on 60 atomic force microscopy (AFM in Figure 7c) measurements at different locations, it turned out that Ag crystallites reached a penetration depth up to 40 ± 5 nm. As shown in Table 4, the emitter thickness of around 200 nm is large enough to avoid substantial recombination due to the metallization.

The most critical factor affecting $V_{oc}$ in this experiment was the doping profile. The largest $V_{oc}$ was obtained for the shallowest profile, regardless of the silver paste. The highest obtained $V_{oc}$ were 657 mV and 655 mV depending on the paste. In all cases, these experimental results support the specified optimal thickness of the doping layer as well as the surface concentration, that is, thinner and lower surface concentrations with respect to the experimental value shown in Table 1.

Another study based on modeling and supported by experimental measurements reported that if the doped layer is etched partially over a large fraction beneath the contact, it is a possible scenario when the glass frit is etching into the emitter during the firing step or when the Ag crystallites cover a larger fraction under the contact. This effect is more significant as the junction depth is reduced, which translates into a shallow doping profile due to the less effective shield for minority charge carriers at the non-passivated surface [47].

## 6. Conclusions

In the present work, a model of a passivated emitter and rear totally diffused (n-PERT) crystalline silicon solar cell was obtained based on the drift-diffusion and continuity equations with the semiconductor module of COMSOL Multiphysics v6. The computational results were in good agreement with the experiments. The model can be used to study a *p*^+^*nn*^+^ structure and, thus, a family of cases, where *p*^+^ refers to the emitter, *n* is the base, and *n*^+^ is the back surface field (BSF). Additionally, the model can work for the case of monofacial and bifacial solar cells under any illumination, which means, only front, only rear, or simultaneous front and rear side illumination.

Applying a genetic algorithm (GA) in MATLAB to the solar cell model, six cell parameters were optimized for obtaining maximum output power when illuminated by a representative spectrum of the Atacama Desert (AM1.08). For comparison, the optimization to the standard reference global solar spectrum AM1.5G was also carried out. The control variables were thickness and doping concentration of the emitter, cell, and back surface field (BSF). Given that the Atacama and reference spectra differ, the optimal parameters of the n-PERT solar cell resulted to be distinct for each illumination. Under AM1.08 illumination, the BSF is required to be thinner and lightly doped compared with values under the AM1.5G spectrum. Moreover, the doping of the emitter needs to be heavier compared to the doping under the AM1.5G spectrum. In summary, the optimized solar cell under the AM1.08 spectrum led to a gain of +4.9% for the short circuit current density *J*_sc_, +5.7% for the power *P*_mpp_, and +5.4% for the efficiency *η*, with a loss in the open circuit voltage *V*_oc_ of 1%.

These results show that considering the shape of the typical local solar spectrum in PV cell design vs. the standard solar spectrum defined under US parameters can improve cell efficiency.

## Figures and Tables

**Table 1:** Input parameters obtained through measurements [17,18,19].

Name	Value	Description
$d_{cell}$	180 µm	Solar cell thickness
$d_{E}$	0.65 µm	Emitter depth
$d_{BSF}$	0.45 µm	Thickness of BSF
$N_{E}$	2.44 × 10^19^ cm^−3^	Emitter surface conc.
$N_{B}$	8.436 × 10^14^ cm^−3^	Base doping
$N_{BSF}$	6.17 × 10^19^ cm^−3^	BSF surface conc.
$\tau_{n}$	1.5 ms	SRH Carrier lifetime
$\tau_{p}$	1.5 ms	SRH Carrier lifetime
$f_{met}$	0.052	Metal fraction front side

**Table 2:** Measured and calculated JV parameters.

	JV Measurement		JV Simulation	
Parameter	Front	Rear	Front	Rear
$J_{sc}\;\left(\mathrm{mA}/{\mathrm{cm}}^{2}\right)$	39.2 ± 0.03	34.6 ± 0.03	39.2	34.2
$V_{oc}\;\left(\mathrm{mV}\right)$	653.1 ± 2	649.7 ± 2	646.4	654.6
$P_{mpp}\;\left(W\right)$	4.9 ± 0.02	4.3 ± 0.09	5.3	4.6
FF (%)	78.3 ± 0.2	78.2 ± 0.16	78.7	78.7
η (%)	20 ± 0.08	17.6 ± 0.1	20.0	18.0

**Table 3:** Thickness and doping parameters of the cell.

Solar Cell Parameters	Initial Values	Range
$d_{E}$ (nm)	650	50–750
$d_{cell}$ (nm)	180 × 103	150 × 103–200 × 103
$d_{BSF}$ (nm)	450	50–750
$N_{E}$ (cm^−3^)	2.44 × 10^19^	1 × 10^19^–1 × 10^20^
$N_{B}$ (cm^−3^)	8.44 × 10^14^	1 × 10^14^–5 × 10^15^
$N_{BSF}$ (cm^−3^)	6.16 × 10^19^	1 × 10^19^–5 × 10^20^

**Table 4:** Parameters of the genetic algorithm (GA).

GA Parameters	Value
Population size	70
Generations	110
Stopping criterium	10
Mutation probability	10%

**Table 5:** Output electrical parameters extracted from the optimized solar cell.

	Non-Optimized		Optimized		Increment	
Parameter	STC AM1.5G	ATA AM1.08	STC AM1.5G	ATA AM1.08	STC AM1.5g	ATA AM1.08
$J_{sc}\;\left(\mathrm{mA}/{\mathrm{cm}}^{2}\right)$	39.2	42.2	40.7	44.3	3.7%	4.9%
$V_{oc}\;\left(\mathrm{mV}\right)$	646.4	647.0	641.7	640.1	−0.7%	−1.1%
$P_{mpp}\;\left(W\right)$	5.3	5.7	5.5	6.1	4.3%	5.7%
FF (%)	78.7	78.9	79.1	80.1	0.5%	1.5%
η (%)	20.0	21.6	20.8	22.7	4.3%	5.4%

**Table 6:** Summary of initial and optimized solar cell parameters.

Description	Parameter	Exp. Values	AM1.5G	AM1.08
Emitter thickness	*d*_E_ (nm)	650	200.2	201
Cell thickness	*d*_cell_ (nm)	180 × 10^3^	154.2 × 10^3^	165.1 × 10^3^
BSF thickness	*d*_BSF_ (nm)	450	330	250
Emitter doping	*N*_E_ (cm^−3^)	2.44 × 10^19^	9.89 × 10^19^	9.36 × 10^19^
Base doping	*N*_B_ (cm^−3^)	8.44 × 10^14^	9.83 × 10^14^	9.81 × 10^14^
BSF doping	*N*_BSF_ (cm^−3^)	6.16 × 10^19^	3.87 × 10^20^	4.92 × 10^20^

**Table 7:** Measured and calculated current density values.

	n-PERT Front (AM 1.5G)	n-PERT Rear (AM 1.5G)	n-PERT Front (AM 1.08)	n-PERT Rear (AM 1.08)
Measured J_sc_ (mA/cm^2^)	39.2	34.6	X	X
Calculated J_sc_ via COMSOL (mA/cm^2^)	39.2	34.2	44.3	X
Calculated J_ph_ (mA/cm^2^)	38.9	34.3	42.2	37.1

## Data Availability

Not applicable.

## Appendix A

In this section, input parameters used for the solar cell simulation are provided.

**Table A1:** Summary of input parameters. Note that the shown $d_{E},\;d_{cell},\;d_{BSF},\;N_{E},\;N_{B},\;\mathrm{and}\;N_{BSF}$ values were used for the mesh and validation study. However, these parameters were control variables in the optimization step.

Input Parameter	Value
Emitter thickness, $d_{E}$ (nm)	650
Wafer thickness, $d_{cell}$ (µm)	180 × 10^3^
BSF thickness, $d_{BSF}$ (nm)	450
Simulated area, A (cm^2^)	1
Relative dielectric constant, $\varepsilon_{r}$	11.7
Electron affinity, χ (eV)	4.05
Bandgap, $E_{g}$ (eV)	1.12
Effective conduction band density, $N_{c}$ (cm^−3^)	(T/300[K]) ^3/2^ × 1.04 × 10^19^ [cm^−3^]
Effective valence band density, $N_{v}$ (cm^−3^)	(T/300[K])^3/2^ × 2.8 × 10^19^ [cm^−3^]
Electron mobility, $\mu_{n}$ (cm^2^V^−1^s^−1^)	Arora model
Hole mobility, $\mu_{p}$ (cm^2^V^−1^s^−1^)	Arora model
Front-side surface doping, $N_{E}$ (cm^−3^)	2.44 × 10^19^
Base doping concentration, $N_{B}$ (cm^−3^)	8.44 × 10^14^
Rear-side surface doping, $N_{BSF}$ (cm^−3^)	6.17 × 10^19^
c-Si density, ρ (kg/m^3^)	2329
Auger Recombination coefficient for electrons, $C_{n}$ (cm^6^s^−1^)	2.80 × 10^−31^
Auger Recombination coefficient for holes, $C_{p}$ (cm^6^s^−1^)	9.90 × 10^−32^
Direct band-to-band recombination coefficient, C (cm^3^s^−1^)	4.73 × 10^−15^
Tau trap for electrons and holes, $\mu_{n},\;\mu_{p}$ (ms)	1

**Table A2:** Summary of input parameters for the Arora model for the mobility of electrons $\mu_{n}$ and holes $\mu_{p}$, taken from [34]. The Arora model equations are shown below the table.

Input Parameter	Value
Electron mobility reference, $\mu_{n,0}$ (cm^2^/(Vs)	1252
Hole mobility reference, $\mu_{p,0}$ (cm^2^/(Vs)	407
Electron mobility reference minimum, $\mu_{n,min}^{ref}$ (cm^2^/(Vs)	88
Hole mobility reference minimum, $\mu_{p,min}^{ref}$ (cm^2^/(Vs)	54.3
Electron reference impurity concentration, $N_{n,0}^{ref}$ (1/cm^3^])	1.26 × 10^17^
Hole reference impurity concentration, $N_{p,0}^{ref}$ (1/cm^3^)	2.35 × 10^17^
Alpha coefficient, $\alpha_{0}$	0.88
Mobility reference minimum exponent, $\beta_{1}$	−0.57
Mobility reference exponent, $\beta_{2}$	−2.33
Impurity concentration reference exponent, $\beta_{3}$	−2.33
Alpha coefficient exponent, $\beta_{4}$	−0.146
Reference temperature, $T_{ref}$ (K)	300

Arora model equations [34,35]:

$$\mu_{n}=\mu_{n,min}+\frac{\mu_{n,0}}{1+{\left(\frac{N}{N_{n0}}\right)}^{\alpha_{n}}}$$

$$\mu_{p}=\mu_{p,min}+\frac{\mu_{p,0}}{1+{\left(\frac{N}{N_{p0}}\right)}^{\alpha_{p}}}$$

$$\mu_{n,min}=\mu_{n,min}^{ref}{\left(\frac{T}{T_{ref}}\right)}^{\beta_{1}}$$

$$\mu_{p,min}=\mu_{p,min}^{ref}{\left(\frac{T}{T_{ref}}\right)}^{\beta_{1}}$$

$$\mu_{n,0}=\mu_{n,0}^{ref}{\left(\frac{T}{T_{ref}}\right)}^{\beta_{2}}$$

$$\mu_{p,0}=\mu_{p,0}^{ref}{\left(\frac{T}{T_{ref}}\right)}^{\beta_{2}}$$

$$N_{n,0}=N_{n,0}^{ref}{\left(\frac{T}{T_{ref}}\right)}^{\beta_{3}}$$

$$N_{p,0}=N_{p,0}^{ref}{\left(\frac{T}{T_{ref}}\right)}^{\beta_{3}}$$

$$N=N_{a}^{-}+N_{d}^{+}$$

## References

[1] Fell A., McIntosh K.R., Altermatt P.P., Janssen G.J.M., Stangl R.A., Ho-Baillie H., Steinkemper J., Greulich M., Muller B., Min B. (2015). Input Parameters for the Simulation of Silicon Solar Cells in 2014. IEEE J. Photovoltaics.

[2] Peng Z.-W., Koduvelikulathu L.J., Kopecek R. The impact of bulk resistivity on bifacial n-PERT rear junction solar cells. Proceedings of the Conference Record of the Photovoltaic Specialists Conference.

[3] Ferrada P., Marzo A., Cabrera E., Chu H., del Campo V., Rabanal J., Diaz-Almeida D., Schneider A., Kopecek R. (2017). Potential for photogenerated current for silicon based photovoltaic modules in the Atacama Desert. Sol. Energy.

[4] Dehghanzadeh A., Farahani G., Maboodi M. (2017). A novel approximate explicit double-diode model of solar cells for use in simulation studies. Renew. Energy.

[5] Boussaid M., Belghachi A., Agroui K., Abdelaoui M., Otmani M. (2016). Solar cell degradation under open circuit condition in out-doors-in desert region. Results Phys..

[6] Vincent P., Sergio G.C., Jang J., Kang I.M., Park J., Kim H., Lee M., Bae J.-H. (2020). Application of Genetic Algorithm for More Efficient Multi-Layer Thickness Optimization in Solar Cells. Energies.

[7] Katoch S., Chauhan S.S., Kumar V. (2021). A review on genetic algorithm: Past, present, and future. Multimed. Tools Appl..

[8] Razzaq A., Mayer A., Depauw V., Gordon I., Hajjiah A., Poortmans J. (2020). Application of a Genetic Algorithm in Four-Terminal Perovskite/Crystalline-Silicon Tandem Devices. IEEE J. Photovolt..

[9] Attari K., Amhaimar L., El Yaakoubi A., Asselman A., Bassou M. (2017). The Design and Optimization of GaAs Single Solar Cells Using the Genetic Algorithm and Silvaco ATLAS. Int. J. Photoenergy.

[10] Fertig F., Nold S., Wöhrle N., Greulich J., Hädrich I., Krauß K., Mittag M., Biro D., Rein S., Preu R. (2016). Economic feasibility of bifacial silicon solar cells. Prog. Photovolt. Res. Appl..

[11] Phillips A.B., Subedi K.K., Liyanage G.K., Alfadhili F.K., Ellingson R.J., Heben M.J. (2020). Understanding and Advancing Bifacial Thin Film Solar Cells. ACS Appl. Energy Mater..

[12] Rodriguez J., Wang E.-C., Chen N., Ho J.W., Li M., Buatis J.K., Nagarajan B., Xu L., Choy W.L., Shanmugam V. (2018). Towards 22% efficient screen-printed bifacial n-type silicon solar cells. Sol. Energy Mater. Sol. Cells.

[13] Marzo A., Ferrada P., Beiza F., Besson P., Alonso-Montesinos J., Ballestrín J., Román R., Portillo C., Escobar R., Fuentealba E. (2018). Standard or local solar spectrum? Implications for solar technologies studies in the Atacama desert. Renew. Energy.

[14] Polo J., Alonso-Abella M., Chivelet N.M., Alonso-Montesinos J., López G., Marzo A., Nofuentes G., Vela-Barrionuevo N. (2019). Typical Meteorological Year methodologies applied to solar spectral irradiance for PV applications. Energy.

[15] Jessen W., Wilbert S., Gueymard C.A., Polo J., Bian Z., Driesse A., Habte A., Marzo A., Armstrong P.R., Vignola F. (2018). Proposal and evaluation of subordinate standard solar irradiance spectra for applications in solar energy systems. Sol. Energy.

[16] Wilbert S., Jessen W., Gueymard C., Polo J., Bian Z., Driesse A., Habte A., Marzo A., Armstrong P., Vignola F. (2017). Proposal and Evaluation of Subordinate Standard Solar Irradiance Spectra with a Focus on Air Mass Effects.

[17] Marzo A., Ballestrín J., Alonso-Montesinos J., Ferrada P., Polo J., López G., Barbero J. (2021). Field Quality Control of Spectral Solar Irradiance Measurements by Comparison with Broadband Measurements. Sustainability.

[18] Gueymard C.A. (2001). Parameterized transmittance model for direct beam and circumsolar spectral irradiance. Sol. Energy.

[19] Gueymard C. (1995). SMARTS: Simple Model of the Atmospheric Radiative Transfer of Sunshine | Grid Modernization | NREL. https://www.nrel.gov/grid/solar-resource/smarts.html.

[20] Kato S., Rose F.G., Sun-Mack S., Miller W.F., Chen Y., Rutan D.A., Stephens G.L., Loeb N.G., Minnis P., Wielicki B.A. (2011). Improvements of top-of-atmosphere and surface irradiance computations with CALIPSO-, CloudSat-, and MODIS-derived cloud and aerosol properties. J. Geophys. Res. Atmos..

[21] Bellouin N., Quaas J., Morcrette J.-J., Boucher O. (2013). Estimates of aerosol radiative forcing from the MACC re-analysis. Atmospheric Chem. Phys..

[22] Remer L.A., Kaufman Y.J., Tanré D., Mattoo S., Chu D.A., Martins J.V., Li R.R., Ichoku C., Levy R.C., Kleidman R.G. (2005). The MODIS Aerosol Algorithm, Products, and Validation. J. Atmos. Sci..

[23] Wang H., Zou X., Li G. (2012). An Improved Quality Control for AIRS Total Column Ozone Observations within and around Hurricanes. J. Atmos. Ocean. Technol..

[24] (2022). ECMWF | ERA Interim, Daily. https://apps.ecmwf.int/datasets/data/interim-full-daily/levtype=sfc/.

[25] Berrisford P., Dee D., Fielding K., Fuentes M., Kallberg P., Kobayashi S.S. (2009). Uppala, The ERA-Interim Archive. https://www.ecmwf.int/en/elibrary/8173-era-interim-archive.

[26] (2022). Giovanni. https://giovanni.gsfc.nasa.gov/giovanni/.

[27] Ferrada P., Rudolph D., Portillo C., Adrian A., Correa-Puerta J., Sierpe R., Del Campo V., Flores M., Corrales T.P., Henriquez R. (2020). Interface analysis of Ag/n-type Si contacts in n-type PERT solar cells. Prog. Photovoltaics Res. Appl..

[28] Edler A. (2014). Development of Bifacial n-Type Solar Cells for Industrial Application. Universität Konstanz. http://nbn-resolving.de/urn:nbn:de:bsz:352-275017.

[29] Edler A., Mihailetchi V.D., Koduvelikulathu L.J., Comparotto C., Kopecek R., Harney R. (2015). Metallization-induced recombination losses of bifacial silicon solar cells. Prog. Photovolt. Res. Appl..

[30] Slooff L., Brockholz B., Verhees W., Veenstra S., Cobussen-Pool E., Kroon J., Bende E. (2012). Determination of the Intrinsic Diode Parameters of Polymer Solar Cells. Energy Procedia.

[31] Green M.A. (1981). Solar cell fill factors: General graph and empirical expressions. Solid-state Electron..

[32] Vasconcelos J., Saldanha R., Krähenbühl L., Nicolas A. (1997). Genetic algorithm coupled with a deterministic method for optimization in electromagnetics. IEEE Trans. Magn..

[33] Altermatt P.P. (2011). Models for numerical device simulations of crystalline silicon solar cells—A review. J. Comput. Electron..

[34] Arora N., Hauser J., Roulston D. (1982). Electron and hole mobilities in silicon as a function of concentration and temperature. IEEE Trans. Electron Devices.

[35] (2021). Semiconductor Module User’s Guide. COMSOL Multiphysics® v. 6.0. COMSOL AB, Stockholm, Sweden. https://doc.comsol.com/6.0/doc/com.comsol.help.semicond/SemiconductorModuleUsersGuide.pdf.

[36] Chowdhury M.M., Debnath B. Approximation of carrier generation rate in common solar cells and studies for optimization of n+p silicon solar cell for AM1.5G and AM1.5D. Proceedings of the 2012 7th International Conference of Electronic and Computer Engineering ICECE.

[37] Cuevas A., Yan D. (2013). Misconceptions and Misnomers in Solar Cells. IEEE J. Photovolt..

[38] Mahajan S., SreeHarsha K.S. (1998). Principles of Growth and Processing of Semiconductors.

[39] Sai H., Sato Y., Oku T., Matsui T. (2021). Very thin crystalline silicon cells: A way to improve the photovoltaic performance at elevated temperatures. Prog. Photovoltaics: Res. Appl..

[40] Brendel R., Queisser H. (1993). On the thickness dependence of open circuit voltages of p-n junction solar cells. Sol. Energy Mater. Sol. Cells.

[41] Sinton R.A., Swanson R.M. (1987). Recombination in highly injected silicon. IEEE Trans. Electron Dev..

[42] Dhariwal S., Kulshreshtha A.P. (1981). Theory of back surface field silicon solar cells. Solid-State Electron..

[43] Stem N., Cid M. (2001). Studies of phosphorus Gaussian profile emitter silicon solar cells. Mater. Res..

[44] Almeida D.D., Ferrada P., Marzo A., Cabrera E., Urrejola E., Espinoza D., Castillo R., Llanos J., Portillo C. Theoretical Calculation of the Photo-generated Current Density by Using Optical Path-length Enhancement Factor for Si-based PV Devices in the Atacama Desert. Proceedings of the 2018 IEEE 7th World Conference on Photovoltaic Energy Conversion, WCPEC.

[45] Diaz Almeida D.F., Araya P., Ferrada A., Sanz Martinez N., Yurrita Zubillaga O. Evaluation of Antireflection and Antisoiling Coatings for PV Modules in the Atacama Desert. Proceedings of the 33rd European Photovoltaic Solar Energy Conference and Exhibition.

[46] Cabrera E., Olibet S., Rudolph D., Glatz-Reichenbach J., Kopecek R., Reinke D., Gotz A., Schubert G. Impact of Si surface topography on the glass layer resulting from screen printed Ag-paste solar cell contacts. Proceedings of the Conference Record of the IEEE Photovoltaic Specialists Conference.

[47] Koduvelikulathu L.J., Mihailetchi V.D., Olibet S., Rudolph D., Cabrera E. (2015). Two-Dimensional Modeling of the Metallization-Induced Recombination Losses of Screen-Printed Solar Cells. IEEE J. Photovolt..

